# A systems approach to personalised nutrition: Report on the Keystone Symposium “Human Nutrition, Environment and Health”

**DOI:** 10.1016/j.atg.2016.08.001

**Published:** 2016-08-04

**Authors:** Michael Maher, Amy M. Pooler, James Kaput, Martin Kussmann

**Affiliations:** Nestlé Institute of Health Sciences, Lausanne, Switzerland

## 1 Introduction

Personalised medicine has emerged as a novel strategy for treating disease. By combining analyses of genetic and environmental factors, treatments can be specifically tailored to the individual, thereby improving their efficacy. This approach has been particularly useful in cancer, where high heterogeneity in tumour phenotypes and microenvironments make “one-size-fits-all” treatments difficult (Block et al., 2015). The success of these personalised approaches suggests that extension to other areas, including both disease prevention and maintenance of good health, will also be fruitful.

Personalised nutrition aims at maintaining and optimising health so as to prevent disease (Kaput et al., 2015b). Current nutritional guidelines are typically derived from epidemiological and associative studies and resulting large clinical databases. Therefore, they are not always useful or actionable for individuals. Indeed, a recent study demonstrated that individuals produce very different changes in blood glucose levels, even after consuming the exact same food (Zeevi et al., 2015). Therefore, in order to help individuals manage e.g. their glycaemic responses to meals, tailored solutions and recommendations must be developed.

Research into personalised nutrition is accelerating, and this approach will be crucial for preventing complicated and highly individualised conditions such as metabolic disease and obesity. Diabetes research is an excellent example of how large-scale analyses have yielded insights into the disease complexity, at the level of both the population and the individual. For example, recent studies have identified numerous genetic risk factors related to Type 2 diabetes (Prasad and Groop, 2015); others are exploring the complicated relationship between genes and environmental factors (Franks and Pare, 2016), as well as epigenetic risk factors (Dayeh et al., 2016). However, understanding the enormous amount of genetic data and highly complex (and largely unknown) diet-gene interactions to design individualised nutritional recommendations poses a significant challenge. Indeed, researchers must analyse and correlate multiple kinds of data, including not just genetics, but also modifications of these genes (epigenetics), blood levels of nutrients, glucose tolerance, and numerous additional parameters. Resolving these issues will require a concerted effort from the fields of genetics and informatics, combining the latest ‘omics’ technologies with the power of big data analytics.

The scope of the Keystone Symposium “Human Nutrition, Environment and Health” in Beijing, China (October 14th to 18th 2015), was to discuss both the opportunities and challenges for personalised nutrition and the application of systems biology to healthcare. The conference was organised by three leading figures in the field of nutritional and systems science, Martin Kussmann (Nestlé Institute of Health Sciences, Lausanne, Switzerland), Hannelore Daniel (Technical University Munich, Germany) and Jacqueline Pontes Monteiro (University of São Paulo, Brazil), together with the Beijing Genomics Institute. This first-of-its-kind Keystone Symposium and unique event brought experts together from academia, public health care, and industry to discuss state-of-the-art research in the nutritional sciences. Participants from 40 different countries joined the meeting, which made for a truly global snapshot of current nutrition research.

The main objective of the meeting was to explore and connect novel quantitative, comprehensive, and molecular approaches to nutrition research, with a focus on personalised nutrition. The talks of the meeting were divided into seven major topics: (1) the interaction between human genome, diet and environment; (2) translational models for human nutrition; (3) human nutritional and lifestyle interventions; (4) capturing and monitoring human individuality; (5) nutrigenomics and systems nutrition; (6) Nutrition 2.0 – translation into solution for human health; and (7) global nutrition and sustainability.

The meeting brought together researchers from distinct scientific fields: nutrition, genomics, physiology, epidemiology, clinical research, analytics, and bioinformatics. These topics provided the foundation for discussions surrounding the current state of nutritional sciences. In his keynote address, José Ordovás from Tufts University, USA, noted that we cannot ignore the connectivity between genetics and environment for studying – and eventually understanding – how to achieve optimal nutrition. He re-introduced the concept of the ‘exposome’ (Wild, 2012). First espoused 10 years ago, it describes everything the individual is exposed to in his or her life that is not genetic. Crucial to personalised medicine is linking environmental factors to disease risk and causation, and as such may be just as significant a factor as the genome in determining phenotype; indeed, nutrition is one of the main exposomes. However, establishing causal relationships between disease and exposure is difficult, requiring interpretation of vast quantities of data.

## 2 Novel quantitative, comprehensive and molecular science approaches to nutrition research

As scientists begin to look past the genome to explain phenotypes, correlation of genetics with relevant outputs from different molecular platforms will be crucial, particularly for uncovering the effects of diet-gene interactions.

Martin Kussmann discussed how his research group has used such multi-faceted approaches to further the understanding of metabolism and the interplay between diet, phenotype (e.g., diabetes and obesity), and genetics. Broad investigative tactics allow for interpretations to be made from longitudinal human studies, which include dietary interventions and challenges to homeostasis. Similarly, Hannelore Daniel presented data from the Human Metabolome Study (HuMet; www.humet-tum.de), which deployed multiple molecular techniques to produce a comprehensive analysis of metabolite changes in healthy young male subjects following a number of highly controlled nutritional interventions. The HuMet study measured a large array of analytes, including lipids, lipoproteins, and amino acids, as well as standard parameters such glucose and lactate (Krug et al., 2012). Underpinning the relevance of such broad-ranging studies are robust statistical tools that generate biologically significant inferences. Marie-Pier Scott-Boyer (COSBI, University of Trento, Italy) highlighted the importance of network-based analysis for better understanding the interplay between micronutrients, particularly cofactor-protein interactions (Scott-Boyer et al., 2016). Such network analyses can be extended to the gut microbiome, which contains an enormous number of microbe species and metabolite variability. Metagenomic analysis has revealed insights into gut microbiota and their complex relationship with body mass, glucose intolerance, and inflammation, as presented by Maria-Carlota Dao (Institute of Cardiometabolism and Nutrition, France). Representatives from two informatics-based companies, Viocare and EdgeLeap, showed how industry is fuelling systems nutrition with developing patient- and consumer-based diet monitoring software (Weiss et al., 2010), and large-scale data integration tools (Derous et al., 2015), respectively. These new tools are needed, as tracking an individual's environmental exposure from the pre-natal development period to adulthood is an obvious logistical challenge.

## 3 Improved study design and better models to understand mechanisms of health and disease

Claudio Franceschi (University of Bologna, Italy) provided a convincing case for the use of centenarians as a model for successful healthy ageing and long life, and to assess genetic and environmental risk factors for age-related conditions such as Type 2 diabetes. In a fascinating study which formed part of the wider NU-AGE diet intervention program involving nonagenarians, healthy centenarians (≥ 100 years of age) and their offspring (Santoro et al., 2014), a wide-ranging set of biochemical and omics analyses were carried out in order to understand the molecular, genetic, epigenetic and microbiotic definition of ageing. At the epigenetic level, the “DNA methylation age” of the centenarians and their offspring was “younger” than their respective chronological age, highlighting that protective mechanisms may be in place.

The interventional prospective cohort study is a traditional method of determining the efficacy of treatments or dietary interventions for metabolic diseases. However, following a sample group of similar individuals may highlight the effects on only one particular population, while not taking into account potential dissimilar effects in other populations or, perhaps more importantly, variations between individuals. Jacqueline Pontes Monteiro challenged such conventional approaches to human studies by highlighting the advantages of using the novel “N-of-1” clinical trial to ascertain individualised micronutrient requirements. Nutritional intake guidelines proposed by government organisations are largely based on averages calculated from whole populations (Monteiro et al., 2015). However, tracking the individual metabolic response to micronutrient intake over time can be more informative for personal requirements, and ultimately lead to a nutritional treatment program. Furthermore, individual genetic and epigenetic variability can account for both the predisposition to disease and the degree of response to a treatment intervention. For this reason, the N-of-1 approach (Kaput and Morine, 2012, Schork, 2015), rather than the traditional case/control design, can be utilised for the development of more targeted and defined treatment strategies based on an individual's needs.

Leroy Hood (Institute of Systems Biology, USA) reiterated the potential benefits of the wider usage of N-of-1 studies as a means to develop personal healthcare towards systems medicine. He advocated the implementation of P4 medicine – predictive, preventive, personalised, and participatory (Hood, 2013) – as a means to reform the current healthcare model, which is reactionary to disease, rather than a predictor thereof. Envisaged in this strategy is a virtual cloud to which data points pertaining to individuals' genomic, transcriptomic and proteomic data will be uploaded at different time points over a person's life. Hood believes that this strategy is achievable in the future with the collaboration of scientists, engineers, health professionals, and industry partners.

## 4 Connect health monitoring with personalised dietary counselling

The strengths and limitations of current health monitoring were discussed by a number of the contributors to the symposium. Web- and mobile phone-based applications for the monitoring of lifestyle and habits, for example, are now in mainstream use among individuals, but they have yet to be implemented in clinical settings on a widespread basis. Much validation remains to be done to bring personalised healthcare to a robust position where it can be applied by health services. Lorraine Brennan (University College Dublin, Ireland) has been involved in such proof-of-concept work. Her research is part of the European Food4Me project (Celis-Morales et al., 2015), which aims to comprehensively determine the feasibility of personalised nutrition on a number of levels; including determining the most suitable genetic and transcriptomic markers of interest; ethical and legal issues surrounding personal health monitoring; and developing novel means of such self-monitoring. Brennan illustrated how innovative metabonomic monitoring of patient health parameters has delivered targeted dietary advice to patients using an online platform for recording and collection of this data (Marshall et al., 2016). Out of curiosity about his own wellness phenotype, Ben van Ommen (Netherlands Organisation for Applied Scientific Research, TNO) went a step further by assuming the role of the patient and performed an N-of-1 study on himself. He showed data from his own self-prescribed dietary and lifestyle intervention during which he monitored the changes in specific biomarkers, using freely available online platforms to record and update his progress, and ultimately improved his health. He demonstrated that as services like this become more widely available, personalised nutrition can enter into conventional practice.

## 5 Insights into nutrition research from across the globe

The environmental impact of modern food consumption is often overlooked, not least in the context of healthy eating. Jim Kaput (Nestlé Institute of Health Sciences, Switzerland) highlighted the importance of using a systems approach for understanding optimal health in individuals through to populations. This approach not only requires bringing together biomedical data, but extending it to agriculture, environment, and economics (Kaput et al., 2015a). Despite the developments made in food technology and greater crop yields being achieved, malnutrition remains to be a huge public health issue for many areas of the world. Robert Zeigler of the International Rice Research Institute in the Philippines illustrated how rice can improve nutrition among undernourished communities through biofortification with essential micronutrients. While the Western world deals with the epidemic of obesity and metabolic disease, it is vital that the nutritional wellbeing of the world's poorest populations is kept at the forefront in the minds of researchers. Likewise, emphasis must continue to be placed on the effects of the climate change on agriculture, upon which the world's food supply ultimately hangs in the balance.

## 6 Conclusion

Wrapping up the meeting, a panel discussion comprising Jim Kaput, Lorraine Brennan, Ben van Ommen, and Claudio Franceschi was held on the final day. During this discussion including lively contributions from the audience, several topics were debated, such as the need for nutritional training for physicians and the role of the food industry in personalised nutrition. Martin Kussmann brought the session to a close by emphasizing that science must counterbalance the overabundance of conflicting information about nutrition in the media by continuing to provide rigorous evidence and sound nutrition advice.

Personalised nutrition may be able to revolutionise the health of individuals. Collecting such data, however, relies on the continued participation of each person and their participation is of course vital to the success of personalised healthcare. Can such a system of reporting be implemented in the public or should it be confined to clinical settings? What motivates someone to maintain interest in reporting their data? Could certain devices assist in this, or even provide gamification in order to maintain participation? In the era of lab-on-a-chip technologies together with mobile, social media platforms, the gathering and collation of such data is certainly achievable, and new strategies should be examined in order to understand how individuals wish to interact with such information, including implementing data driven nutritional advice. By enabling better understanding of nutritional needs at both the individual and population levels, systems nutrition will empower people across the world to optimise their health.
